# Catch me if you can: how AML and its niche escape immunotherapy

**DOI:** 10.1038/s41375-021-01350-x

**Published:** 2021-07-23

**Authors:** Sarah Tettamanti, Alice Pievani, Andrea Biondi, Gianpietro Dotti, Marta Serafini

**Affiliations:** 1 Tettamanti Research Center, Department of Pediatrics, University of Milano-Bicocca/Fondazione MBBM, Monza, Italy; 2 Lineberger Comprehensive Cancer Center, University of North Carolina at Chapel Hill, Chapel Hill, NC USA

**Keywords:** Cancer microenvironment, Cancer immunotherapy, Acute myeloid leukaemia, Immunosurveillance

## Abstract

In spite of the remarkable progress in basic and preclinical studies of acute myeloid leukemia (AML), the five-year survival rate of AML patients remains poor, highlighting the urgent need for novel and synergistic therapies. Over the past decade, increased attention has been focused on identifying suitable immunotherapeutic strategies for AML, and in particular on targeting leukemic cells and their progenitors. However, recent studies have also underlined the important contribution of the leukemic microenvironment in facilitating tumor escape mechanisms leading to disease recurrence. Here, we describe the immunological features of the AML niche, with particular attention to the crosstalk between the AML blasts and the cellular components of the altered tumor microenvironment (TME) and the mechanisms of immune escape that hamper the therapeutic effects of the most advanced treatments. Considering the AML complexity, immunotherapy approaches may benefit from a rational combination of complementary strategies aimed at preventing escape mechanisms without increasing toxicity.

## Introduction

The concept of adoptive immunotherapy was described for the first time by Mathé in the late ‘60s in the context of allogeneic hematopoietic stem cell transplantation (HSCT) for the treatment of acute myeloid leukemia (AML). The graft-vs-leukemia effect, by which leukemia can be eradicated by the immune cells from the donor graft, revolutionized the field of cancer therapy and has since evolved to targeted immunotherapeutic strategies such as chimeric antigen receptor (CAR) redirected T cells, bispecific T-cell engagers (BiTEs) and checkpoint inhibitors. Furthermore, it became evident that AML development and progression are associated with dysregulated immune responses. In particular, recent studies have highlighted how leukemic cells manipulate and alter the tumor microenvironment (TME) creating a unique niche that directly promotes their survival as well as drug resistance.

This review is aimed at describing the biological properties of the AML niche, the crosstalk between AML blasts, and cellular components of the TME, considering the non-hematopoietic participants such as stromal cells and vascular endothelium, and the development of resistance towards immunotherapeutic strategies. We will then review the most innovative concepts supporting therapeutic combinations that may overcome the current barriers in AML treatment.

## How AML and its niche affect immunotherapy

### AML blast-induced resistance to immunotherapy

**Fig. 1:**
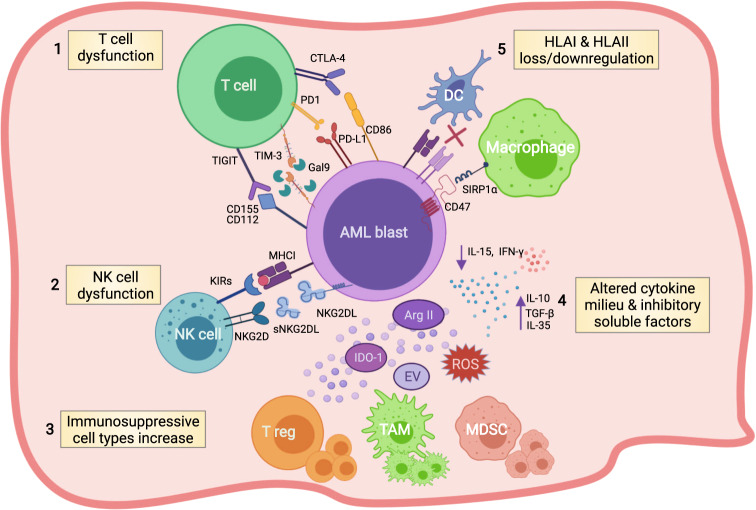
AML blast’s immune escaping strategies. Schematic illustration summarizing the most known immune evasion mechanisms exerted by AML blasts. AML blasts can hamper T- and NK-cell effector functions by aberrantly overexpressing inhibitory T-cell ligands (i.e. PD-L1, Gal-9, CD155, CD112, CD86) (1), or by releasing soluble forms of NKG2DL (2). AML blasts promote T-cell exhaustion and apoptosis, drive the expansion of regulatory T cells (Tregs) and myeloid-derived suppressor cells (MDSCs), and induce the switch of macrophages to tumor-associated macrophages (TAMs) (3) by altering the cytokine milieu and through the release within the BM niche of other soluble factors such as reactive oxygen species (ROS), indoleamine 2,3-dioxygenase-1 (IDO1), arginase II (ArgII), and extracellular vesicles (EVs) (4). Moreover, AML blasts reduce their expression of antigen presentation molecules, thus hiding themselves from immune cells such as dendritic cells (DCs) and macrophages (5). Figure created with Biorender.

There is compelling evidence that AML blasts play a role in the creation of weathered gears in the host immune system through several unique immune evasion mechanisms (Fig. 1). Tumor escape strategies in AML involve direct adaptation of the AML cells to hide from immune recognition and tumor-cell-mediated modifications of the immune cell compartment that include effector T cells, natural killer cells (NKs), and dendritic cells (DCs). With the advent of spatially-resolved immunohistochemistry, high-throughput single-cell transcriptomic, proteomic, and mass cytometry technologies it is possible to better decipher the AML immunologic microenvironment and to envision more tailored immunotherapeutic strategies for the future of AML treatment [1–5].

### AML are defective in antigen presentation

The success of allogenic HSCT relies on the ability of T and NK cells to recognize and eliminate leukemic cells. AML blasts have developed immunoediting processes, such as genetic deletion of HLAs, especially in the context of the haploidentical transplant, and epigenetic downregulation of HLA class II molecules in different donor transplant settings, which preclude conventional recognition of AML blasts by CD8 and CD4 T cells via αβTCR engagement with peptides presented in class I and II. Gene expression profiling of AML blasts from patients relapsed after HSCT has uncovered transcriptional signatures enriched in altered immune-related processes, including the epigenetic downregulation of HLA class II genes, the genomic loss of HLA, the epigenetic upregulation of T cells inhibitory ligands, and the deregulated release of immunosuppressive molecules [6–9].

### AML blasts express immune checkpoint markers

Immune Checkpoints (ICs) are regulatory molecules expressed on T cells to activate self-tolerance and prevent autoimmunity. To evade immune surveillance, leukemic blasts aberrantly express the ligands for ICs [6]. The role of each co-inhibitory pathway in AML has been extensively described in a recent review by Taghiloo and Asgarian-Omran [10]. The most well-known AML ligand for a IC is programmed-cell-death ligand-1 (PD-L1) that, once recognized by the PD-1 receptor on T cells, provides a co-inhibitory signal that causes T-cell exhaustion. Moreover, the PD-1/PD-L1 axis can also promote the expansion of regulatory T cells (Tregs), which further hampers the effector function of CD8 T cells. T-cell immunoglobulin and mucin domain 3 (TIM-3) is a well-defined IC in both effector T and NKcells. TIM-3 binds to galectin-9, which is highly expressed on AML blasts, and has been found to promote self-renewal via stimulatory β-catenin and NFκB-signaling, and to reduce the release of pro-inflammatory cytokines, ultimately resulting in NK- and T-cell dysfunction. Furthermore, galectin-9 is highly involved in creating an autocrine loop that seems essential in the maintenance of leukemic stem cells (LSCs) [11, 12]. In AML murine models and in patients there is a strong association between high frequency of TIM-3^+^ and PD-1^+^ T cells and poor prognosis [13–16]. Another inhibitory receptor, TIGIT (T-cell immunoglobulin and ITIM domain), which binds to the same ligands as DNAM-1, CD155, and CD112, has also been shown to be upregulated in AML blasts. Interestingly, low levels of DNAM-1 expression are observed in AML patients, while its ligands are highly expressed [17, 18] suggesting that the binding of TIGIT with CD112 and CD155 ligands may represent a mechanism of tumor immune escape promoted by immune cell inhibitory signaling. This notion is further supported by clinical observations showing that CD112 and CD155 expression are associated with poor prognosis in AML [19]. In another recent study, high mRNA levels of the inhibitory receptors Cytotoxic T-lymphocyte associated protein 4 (CTLA4) and lymphocyte activating-3 (LAG-3) in AML blasts were also shown to be predictive of an unfavorable prognosis [20]. A peculiar checkpoint and AML LSC marker that plays a crucial role in immune evasion is CD47, a transmembrane protein that by binding to its receptor Signal Regulatory Protein Alpha (SIRPα) on macrophages exerts a “don’t eat me” signal by blocking macrophage engulfment [21]. It is physiologically expressed by various types of normal cells but has emerged as a potent common signal by which cancer cells evade the innate immune system [22].

Immune Checkpoint Inhibitors (ICIs) targeting PD-1/PD-L1 and CTLA4 are highly effective in patients with solid tumors characterized by high neoantigen loads such as melanoma and lung cancer, and have become standard of care for these patients. ICIs are currently explored in multiple clinical trials of patients with hematologic malignancies including AML. Combinatorial approaches are also underway by exploiting other ICIs, such as CD47, TIM-3, TIGIT, and LAG-3.

### AML blasts alter the formation of T-cell immune synapses

One of the most comprehensive study that analyzed the T-cell compartment in AML was performed by Le Dieu et al. They observed that the absolute number of T cells in the peripheral blood (PB) of newly diagnosed AML patients was increased compared with age-matched healthy controls. Furthermore, gene expression profiling revealed an aberrant T-cell activation signature in AML patients. In particular, differentially expressed genes were involved in actin cytoskeletal formation, and correlative functional data demonstrated an impaired capacity of T cells in forming an immune synapse with AML blasts [23]. These results are in line with previous studies showing that T cells isolated from AML patients are phenotypically effector cytotoxic T lymphocytes, express activation markers, but are impaired in their cytotoxic potential defined as the capacity to express cytotoxic granules [24].

### AML secrete immunoinhibitory soluble factors altering T-cell immune responses

T-cell functional alterations in AML are also a consequence of a dysregulated cytokine network directly mediated by AML blasts. Several studies have documented high numbers of Tregs in patients with AML [25, 26]. In particular, Shenghui et al. showed that elevated frequency of CD4^+^CD25^+^CD127^low/-^ Tregs in AML is associated with poor prognosis. Moreover, it was observed that bone marrow (BM)-resident Tregs were more immunosuppressive than Tregs detected in PB further supporting the concept that the AML niche is characterized by multiple layers of inhibitory cells [27]. Tregs enrichment in the AML niche has been associated with the capacity of AML blasts to secret immunoinhibitory factors, such as IL-10, IL-35, transforming growth factor-beta (TGF-β), and indoleamine 2,3-dioxygenase 1 (IDO1) [28–30]. These soluble factors push T-cell polarization towards induced Tregs promoting T-cell tolerance and leukemia progression. IDO1 in particular has been shown to correlate with poor prognosis [31]. IDO1 catabolizes the degradation of tryptophan to N-formylkynurenine. The reduction in local tryptophan concentration and accumulation of toxic tryptophan metabolites cooperate to arrest T-cell proliferation. Moreover, tryptophan-derived metabolites like L-kynurenine inhibit antigen-specific T-cell proliferation and induce T-cell apoptosis. This cytokine imbalance reduces the production of pro-inflammatory cytokines, such as IL-15 and interferon-gamma (IFN-γ), further propagating the negative effects on T-cell effector functions. A more detailed understanding of the altered cytokine profile in AML has been reviewed by Binder et al [32].

Other soluble factors related to different metabolic pathways have also been described to modulate the TME in leukemia. High levels of arginase II in plasma of AML patients were shown to impair T-cell proliferation and to polarize monocytes toward an immunosuppressive M2-like phenotype. In addition, increased arginine metabolism inhibited the proliferation of hematopoietic progenitors, contributing to a wider suppressive TME [33]. Together with arginine II, upregulation of the inducible nitric oxide synthase (iNOS) by AML blasts correlated to inhibition of T-cell proliferation, increase in Tregs, and decreased number of NKT cells [34]. Among the most recently studied metabolites in the tumors are fatty acids and lipid mediators derived from fatty acids. Emerging data suggest that targeting lipid pathways may restore an active immune milieu in AML [35]. In particular, AML blasts can metabolize both glucose and fatty acids, released by surrounding stromal adipocytes, to derive acetyl-CoA to drive the Krebs cycle and oxidative phosphorylation (OXPHOS) for ATP production. Notably, LSCs in the AML niche express the fatty acid transporter CD36, and induce lipolysis in BM adipocytes to fuel fatty acid oxidation (FAO) in leukemic cells [36].

### AML blasts escape NK cell recognition

NK-mediated tumor recognition is MHC-independent and is governed by the interaction of inhibitory and activating receptors on NKs and several ligands expressed on the surface of tumor cells. The graft-versus-leukemia effect mediated by alloreactive NKs in patients receiving haploidentical HSCT represented one of the first evidence that NKs can target and kill residual AML blasts. However, mechanisms of NK-cell evasion and escape by AML blasts have been documented and include an altered expression of NK-cell ligands caused by epigenetic changes, such as incorrect hypermethylation of genes encoding ligands for the activating receptor NKG2D (NKG2DL), namely MICA, ULBP1, ULBP2, and ULBP3 genes [37]. Notably, NKG2DL-negative leukemic cells that escaped the NK-cell immune recognition were shown to have an immature morphology and molecular and functional stemness characteristics, further indicating how AML LSCs and immune evasion are intertwined [38]. Moreover, AML blasts were shown to release a soluble form of NKG2DL (sNKG2DL), through cleavage by metalloproteases or into exosomes, causing the downregulation of NKG2D receptor on NKs and impairing their cytotoxic activity. AML blasts express high levels of ligands, such as CD112 and CD155, that cause a decrease in their activating receptor DNAM-1 on NKs, ultimately leading to an altered degranulation of NKs and impaired cytotoxic activity [39, 40]. AML blasts may also escape NKs by induction of co-inhibitory receptors in NKs that include TIGIT, which inhibits IFN-γ release [41]. High TIGIT expression at engraftment has been associated with a reduced number of NKs in the BM, reduced incidence of acute graft-versus-host disease, and poor survival [42].

### AML blasts increase myeloid-derived suppressor cells and tumor-associated macrophages

Myeloid-derived suppressor cells (MDSCs) cause T-cell tolerance through multiple mechanisms that include expression of V-domain Ig suppressor of T-cell activation (VISTA), PD-L1, IDO1, arginase, and production of reactive oxygen species (ROS), peroxynitrate, and multiple cytokines (TGF-β and IL-10) [43]. AML blasts can promote MDSCs expansion by releasing extracellular vesicles (EVs) containing the oncoprotein MUC1, which, in turn, increases c-myc expression in EVs through microRNA miR34a, leading to MDSCs proliferation [44]. Recently, the Akt/mTOR pathway has been shown to play a critical role in the AML-EV-induced phenotypical and functional transition from monocytes to MDSCs. Monocytes engulfing AML-derived EVs acquire the typical CD14^+^HLA-DR^low^ inhibitory phenotype and upregulate expression of genes characteristic for MDSCs, such as S100A8/9 and cEBPβ [45]. In AML patients, it has been reported that MDSCs were more abundant in BM and in PB compared with healthy controls [44]. There is also an association between Tregs and MDSCs numbers in myelodysplastic syndrome, which correlates with a higher risk of transformation to AML, indicating a potential role for MDSCs in AML progression [46].

Macrophages are critical cellular components of the immunosuppressive TME. The intrinsic plasticity of macrophages renders this cell subset particularly susceptible to tissue-specific regulation. Within the TME, tumor-associated macrophages (TAMs) are generally defined as M2 macrophages, and are characterized by anti-inflammatory activity by secreting arginase, metalloproteinases, TGF-β, IL-10, and other cytokines that cause immune suppression, angiogenesis and tissue repair [47]. Al-Matary et al. reported that TAMs are elevated in the BM of AML patients compared to healthy donors. Moreover, AML blasts can directly drive TAMs to an M2-like phenotype in the BM and spleen of tumor-bearing mice [48].

### Stromal and vascular niches promote resistance to immunotherapy

**Fig. 2:**
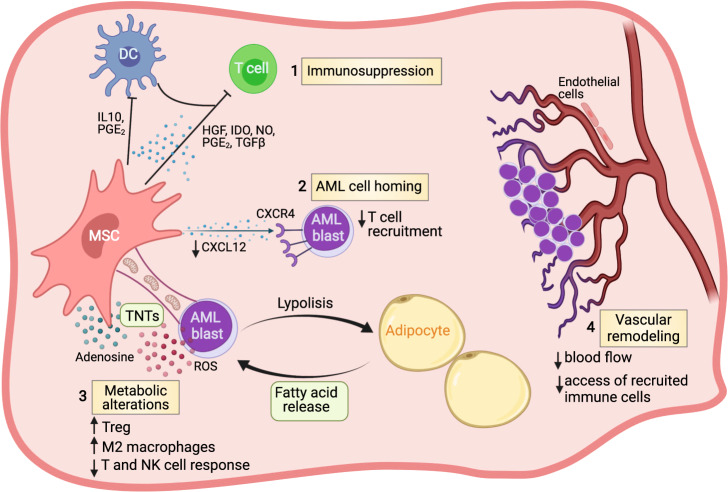
Contributions of non-hematopoietic cells in the bone marrow niche to AML immune escape. Representative mechanisms by which AML-reprogrammed niche cells can promote immune evasion. Mesenchymal stromal cells (MSCs) can regulate the immune response in the leukemic BM microenvironment by secreting a plethora of inhibitory factors, as soluble molecules or as a component of exosomes (1). These factors can inhibit cell proliferation, cytolysis, and production of anti-leukemia cytokines by effector lymphocytes. MSCs mainly through the altered production of CXCL12, interacting with CXCR4, can influence the fate of leukemic cells by triggering their proliferation, spread, and survival and regulate leukocyte migration to the BM niche (2). MSCs and adipocytes regulate the leukemia cells’ metabolism (3). The mitochondrial transfer has recently been appreciated to be a mechanism of intercellular communication associated with chemoresistance and potentially also with immune resistance. Tunneling nanotubules (TNTs) appear to be the primary exchange route used by MSCs to donate mitochondria to AML blasts, boosting oxidative phosphorylation and consequently ROS production which is used by AML blasts as a strategy to evade anti-leukemic effector lymphocytes. A reciprocal relationship occurs between AML blasts and adipocytes wherein malignant cells induce lipolysis from adipocytes and, in turn, adipocytes release fatty acids, which are used as an energy source by malignant hematopoietic cells. Fatty acid oxidation seems to promote the development and activity of immunosuppressive immune cells, such as Tregs and M2 macrophages. The dramatic increase in BM vascular permeability and decrease of blood flow that coincides with leukemic growth can alter the ability of T cells to home, adhere, and extravasate into the leukemic reservoir (4). Figure created with Biorender.

The TME in AML causes resistance to conventional chemotherapy and suppresses anti-tumor immune responses. In fact, leukemia-associated remodeling within the AML niche, including changes associated with increased hypoxia and inflammation as well as metabolic reprogramming, facilitate immune evasion and activation of survival pathways favoring AML progression (Fig. 2) [49].

### Immunosuppressive properties of mesenchymal stromal cells in AML

Mesenchymal stromal cells (MSCs) physiologically display a unique immune regulation ability by inhibiting/reprogramming the activation, proliferation, and function of both adaptive and innate immune cells, as extensively recently reviewed [50]. The role of MSCs in inhibiting innate and adaptive immunity in hematological malignancies, and in particular in AML, is under active investigation. Vasold and colleagues have reported that AML blasts cultured with MSCs are less susceptible to NK cell-mediated killing and that the stromal-induced protection in AML was cell-cell contact-dependent [51]. Toll-like receptor (TLR) 4 may contribute to the MSC-induced inhibition of NK cell function. Sorted TLR4^+^ MSCs derived from AML patients decreased NK cell proliferation and cytotoxicity, and this effect was enhanced by the activation of the TLR4 pathway following lipopolysaccharides treatment [52, 53].

The inhibitory effect of MSCs isolated from AML patients is further supported by their capacity to induce Tregs and upregulate IDO1 [54, 55]. Similarly, MSCs isolated from patients with myelodysplastic syndrome inhibit DC functions through increased production of TGF-β [56]. Other studies reported that AML-derived MSCs are more immunosuppressive and anti-inflammatory than MSCs isolated from healthy donors, showing enhanced suppression of lymphocytes proliferation in vitro and diminished secretion of pro-inflammatory cytokines such as IL-10 [57]. It has also been shown that different clinical/cytogenetic AML subgroups may associate with different profiles of MSCs. Lopes et al. reported that MSCs in AML patients at diagnosis are characterized by high levels of vascular endothelial growth factor A (VEGFA), chemokine ligand 12 (CXCL12), prostaglandin E2 (PGE_2_), IDO1, IL-1β, IL-6, and IL-32 and decrease of IL-10 compared to MSCs collected at the time of disease relapse indicating that MSCs as TAMs are plastic cells that may respond to environmental stimuli [58].

Immunosuppression mediated by MSCs can be further exacerbated by the capacity of AML blasts to generate an inflamed TME. Thus, modifications of the inflammatory status induced by therapy can reshape the immunomodulatory activity of MSCs. In patients with juvenile myelomonocytic leukemia, MSCs showed differential mRNA expression, including genes involved in immunomodulation, that normalized when disease remission was achieved after HSCT [59]. If MSCs can affect immune-based therapies remains to be elucidated. The cytokine storm observed in patients treated with CAR T cells can potentially elicit the immunosuppressive activity of MSCs and cause cell death of MSCs, which is required for triggering their immunosuppressive effects [60]. Recent data showing that MSCs inhibit T-cell responses but do not compromise CD19-specific CAR T cell activity seems indicating that the potent effector function of CAR T cells may overcome the inhibitory effects of MSCs, but additional studies are needed to mechanistically highlight this phenomenon [61].

### AML niche shows altered immune cell homing

CXCL12 expressed by BM stromal cells and its receptor CXC receptor 4 (CXCR4) play a key role in the migration of LSCs to the BM niche. High expression of CXCR4 on AML blasts has been shown to predict poor prognosis [62]. The CXCL12/CXCR4 axis can also activate pathways that favor the survival, growth, and chemotherapy resistance of AML blasts [63]. CXCL12 expression seems to be reduced in MSCs in AML, fostering the migration of CXCR4-overexpressing malignant LSCs *versus* normal hematopoietic stem cells (HSCs) [64]. CXCR4 is also involved in the trafficking of adoptively transferred lymphocytes or CAR T cells to the BM niche. Suppression in the ability of stromal cells to produce CXCL12 in the AML TME may dampen their migration and infiltration into the BM, as reported for other hematological malignancies [65].

### Metabolic alterations of the AML niche

It has been established that leukemic cells present deregulated energy metabolism, which may be involved in causing immune evasion. Competition for critical nutrients such as glucose or amino acids increased release of bioactive inhibitory metabolites such as ROS, and overall microenvironmental metabolic remodeling in tumors including AML have been reported playing inhibitory effects on the immune cell subsets [66]. MSCs are capable of further triggering these metabolic alterations by increasing the bioavailability of nutrients or by the direct transfer of key machineries between cells. MSCs from AML patients have a higher propensity to differentiate into adipocytes, and the interaction between AML blasts and adipocytes in the BM niche creates a unique microenvironment that supports the metabolic demands of leukemia [36, 67]. AML blasts induce hormone-sensitive lipase in adipocytes and activate lipolysis, which then enable FABP4-dependent transfer of fatty acids to leukemia cells, thus enhancing FAO [68, 69]. Fatty acid abundance can hamper effector T-cell functions and promote Tregs differentiation [70]. In fact, FAO can inhibit the activation of effector T cells by increasing PD-1 expression and inhibiting INF-γ secretion, while promoting Treg cell generation through activation of the MAPK signaling pathway. Moreover, FAO has also a key role in polarizing M2 macrophages [71].

MSCs can transfer mitochondria to AML cells through endocytic pathways or tunneling nanotubes (TNT), a process that is further boosted by chemotherapy and associated with increased oxidative phosphorylation-derived ATP production in the recipient cells [72]. AML-derived nicotinamide adenine dinucleotide phosphate oxidase-2 (NOX2) drives the transfer of mitochondria via the generation of superoxide [73]. Recently, gap–junction interactions between AML cells and MSCs in the leukemic niche have been implicated in the regulation of leukemic cell metabolism [74]. The constitutive activation of NOX and the mitochondrial production linked to OXPHOS are the primary sources of large amounts of ROS that are particularly abundant in AML of M4 and M5 subtypes [75, 76]. AML blasts can use ROS to evade anti-leukemic effector lymphocytes since free radicals inactivate T and NK cells by triggering PARP-1 dependent apoptosis [77].

### Vascular niche remodeling in AML

Solid tumors undergo significant remodeling of the blood vessels, which hinders the efficient recruitment of T cells to the tumor site [78]. Furthermore, hypoxia secondary to poor perfusion contributes to inhibit T and NK cells through the activation of their adenosine A2 receptors by the abundant adenosine present in the hypoxic environment [79]. Indeed, extracellular ATP is markedly increased in the AML niche [80] and it is transformed in the immunosuppressive mediator adenosine by the ectoenzymes CD73 and CD39 expressed on tumor cells, Tregs and MDSCs [81]. AML progression has been shown to cause significant remodeling of vascular endothelium mainly via nitric oxide (NO), with increased vascular permeability and decreased blood flow, which results in the formation of a hypoxic leukemia niche [82]. The endosteal BM region is the main site of this vessel loss [83]. As a consequence, several BM areas are hypoperfused and both drug biodistribution and immune cell trafficking are compromised [84, 85]. Finally, the adhesive properties of the immune cells to the endothelium are also altered due to the increased levels of E-selectin induced by the inflammation generated by AML blasts [86].

## Combinatorial strategies to overcome AML resistance to immunotherapy

The complex immunosuppressive cell network of the AML niche can affect even the most advanced immune-based therapeutic strategies, such as ICIs and CAR T cells. Combinatorial strategies aimed at overcoming multiple immunosuppressive mechanisms, as well as at targeting non-malignant components of the TME, such as stromal cells and vascular endothelium, may represent a novel way to enhance immunotherapy effectiveness.

### Targeting AML by combinatorial strategies

#### Improving BiTEs or DART anti-leukemic activity

An illustrative clinical experience of AML escape from immunotherapeutic approaches relates to the studies with the bispecific T-cell engager (BiTE®) antibody construct CD3xCD33, AMG330. BiTEs are bispecific antibody-based molecules composed of two single-chain fragment variable (scFv) domains derived from two different antibodies, one specific for a tumor-associated antigen and the other one for CD3ε, on one polypeptide chain [87]. Treatment with AMG330 showed that the simultaneous engagement of target cells (CD33^+^ AML blasts) and effector cells (CD3^+^ T cells) facilitated recruitment and expansion of effector T cells leading to the elimination of AML blasts even at very low effector-to-target ratios of up to 1:80 in vitro. However, engaged CD4^+^ and CD8^+^ memory T cells upregulated PD-1, TIM-3, and LAG-3, indicating that these cells remain susceptible to checkpoint inhibition [88]. PD-1/PD-L1 blockade led to a significant increase of AMG330-mediated lysis, T-cell proliferation, and IFN-γ secretion [89]. In a reported clinical study, 8 of 42 evaluable patients responded to AMG330 and preliminary response assessment showed that patients with high tumor burden had decreased response to AMG330 [90]. In search for other factors that might cause resistance to AMG330, Harrington et al. found that a favorable response correlated with the number of endogenous T cells, while the levels of CD33 in AML blasts, disease-risks and drug resistance did not correlate with responses [91]. The positive effects of the PD-1/PD-L1 have also been observed in studies using FLT3 BiTE, AMG427 (NCT03541369) [92].

DART, or dual-affinity retargeting antibodies, are bispecific antibodies that have increased stability and half-life compared to BiTEs. In particular, flotetuzumab, a CD123xCD3 bispecific DART, showed encouraging activity in AML patients with an objective response rate ranging between 18% and 30%, but also increased incidence of cytokine release syndrome [93]. Of note, patients with early disease progression showed higher baseline levels of PD-L1 on AML blasts [94].

#### CAR T cells in AML

The success of CAR T cell therapy in B cell malignancies has yet to be realized in AML. In addition to the identification of the most appropriate target in AML to maximize efficacy, but preventing myeloid suppression, preclinical evidence suggests that CAR T cells may be more susceptible to checkpoint inhibition in AML compared to B cell malignancies. Kenderian et al. found PD-1 and TIM-3 pathways to be involved in CAR T cell loss of function in AML. Incubation of primary AML samples with CD33- or CD123-redirected CAR T cells resulted in a significant upregulation of PD-L1 on AML blasts and the combination of CAR T cells with ICIs increased the anti-tumor effects of CAR T cells [95]. Our group has also recently observed upregulation of PD-1 and TIM-3 on cytokine-induced killer cells (CIK) expressing a CD33-specific CAR isolated from the BM of tumor-bearing mice nonresponding to the treatment [96].

In addition to ICIs [97–99], epigenetic drugs can also be used in the attempt to restore AML ligands [37] and T-cell functions [100, 101] within the immunosuppressive TME [102]. CAR T cells combined with ICIs are currently under preclinical and clinical investigation for the treatment of both solid and hematological tumors, as recently reviewed by Hosseinkhani N et al. [103]. Novel bispecific CAR T cell constructs targeting both CD13 and TIM-3 have shown eradication of AML cells in xenograft models. Bispecific CAR T cells showed lower PD-1 and TIM-3 expression in the BM, suggesting that TIM-3 targeting may have a potential immunomodulatory effect [104]. Baragaño Raneros A et al. observed that DNA methylation can contribute to the absence of NKG2DL expression during AML development. Treatment with inhibitors of histone deacetylase (HDACi) and DNA methyltransferase (DNMTi) was found to restore NKG2DL (MICA and ULBPs 1–3) expression in AML through the hypomethylation of *TIMP3*, an inhibitor of protease ADAM17, the sheddase involved in the release of soluble NKG2DL by AML cells [105]. The DNA-demethylating agent decitabine (DAC) has been recently shown to significantly enhance anti-leukemia functions of CD123-specific CAR T cells in vitro and in vivo. Transcriptomic profiling revealed that DAC treatment conferred to CD123 CAR T cells an enrichment of genes associated with naive, early memory, as well as non-exhausted T cells [100]. Moreover, the next-generation CAR design is conceived to boost the anti-tumor function by using immune agonist [106] or co-stimulatory cytokines [107, 108], and to skew T-cell phenotype toward a stem cell/central memory state [109–111]. In a recent preclinical study, Ataca Atilla et al. incorporated transgenic IL-15 to enhance the anti-AML activity of CLL-1 CAR T cells. Unexpectedly, and in contrast to the safety observed with CAR T/IL15 combination in other disease settings, IL-15-expressing CLL-1 CAR T cells induced a severe and atypical form of cytokine release syndrome (CRS), associated with high levels of circulating tumor necrosis factor-alpha (TNF-α). Combination of TNF-α blockade and elimination of CAR T cells using an inducible safety switch controlled the adverse events [112]. This study suggests that combinatorial strategies with CARs improve the anti-tumor effects in AML, but a careful evaluation is fundamental to gain efficacy, whilst avoiding toxicity.

### Targeting the leukemic niche to improve the efficiency of immunotherapies

The central role of stromal cells in the regulation of anti-tumor immune response stimulates the development of novel therapeutic strategies that target not only tumor cells directly, but also non-malignant cells contributing in shaping the TME.

Targeting of MSCs can be obtained using cytotoxic drugs or drugs interfering with their immunosuppressive properties. Tyrosine kinase inhibitors can inhibit both growth and function of MSCs [113]. However, MSCs are involved in many physiological processes in the BM (e.g. HSCs maintenance and regulation) and in other organs (maintenance of the structural architecture), and depletion of MSCs may have serious side effects. How normal MSCs differ from leukemia-associated MSCs remains to be determined, and it is critical in guiding the development of specific drugs. In solid tumors, the fibroblast activation protein (FAP), a member of the serine protease family, is expressed by tumor-associated fibroblasts at higher levels than on resident fibroblasts in healthy tissue. Thus, FAP has been considered as a suitable target to eliminate tumor-associated fibroblasts. In particular, FAP-specific CAR T cells have been used to reduce tumor-cell growth, with minimal off-tumor toxicity [114]. However, the relationship between activated fibroblasts and BM MSCs remains unclear [115, 116]. Many small-molecule inhibitors targeting IDO1, heme oxygenase-1 (HO-1), hepatocyte growth factor (HGF), arginase I and II, PGE_2_, and TGF-β [117–119] and MSC immunomodulatory such as aminobiphosphonate zoledronate [120] have been developed opening the use of these drugs in combination with ICIs and adoptive cell therapies.

The mobilization of leukemic cells from the protective BM niche is considered a promising strategy to increase their susceptibility not only to conventional chemotherapeutic agents but also to immunotherapies [121]. Small-molecule inhibitors, short peptides, and antibodies have been developed to disrupt the CXCL12/CXCR4 axis that releases AML blasts from the BM [63], and recent findings suggest that CXCR4 antagonism can potentially synergize with immunotherapies in several clinical trials involving solid tumors.

Metabolic alterations of the leukemic niche are also potentially druggable. Targeting the fatty acid metabolism using FAO inhibitors may increase not only the efficacy of chemotherapeutic agents, as shown by Farge et al. by combining etomoxir and cytarabine [122], but also boost adoptively transferred T cells [71]. Although etomoxir is no longer used clinically due to its side effects [123], other FAO inhibitors, including avocatin B, exhibit similar inhibitory effects on leukemia cell metabolism [124]. The conversion of ATP in adenosine in the AML microenvironment limits anti-tumor immunity through the suppression of multiple immune subsets including T cells. Indeed, genetic depletion of the adenosine 2A receptor which is over-expressed by activated CAR T cells has been found to enhance their anti-tumor function [125, 126].

Commonly used chemotherapeutics such as cytarabine, etoposide and doxorubicin are agents that promote mitochondria uptake by AML cells [75]. Consequently, resistant clones could have increased oxidative metabolism and produce a large amount of ROS that can, in turn, inactivate T cells and NKs. Several approaches have been used to disrupt mitochondria transfer by blocking TNTs, endocytosis, or superoxide. The surface molecule CD38 is critical for the transport of mitochondria from MSCs to AML cells [127]. Daratumumab, a monoclonal anti-CD38 antibody approved for the treatment of multiple myeloma, was shown to block the delivery of mitochondria to AML cells, decrease the oxygen consumption rate, and inhibit the growth of leukemic cells [128, 129]. Main regulators of mitochondrial biogenesis and activity including PGC-1α and NOX2 are also promising targets. Marley et al. showed that inactivation of PGC-1α by knockdown or by reduction in superoxide levels with N-acetylcysteine impaired mitochondrial transfer [73, 130]. Gap junctions are also shown to mediate mitochondria transfer of MSCs. Blocking connexin-43 gap–junction formation had no effect on cytoplasmic transfer, but reduced mitochondria transfer [131].

Finally, remodeling the dysfunctional tumor vasculature in AML and reversing hypoxia may increase drug delivery and enhance T-cell function. In this sense, NOS inhibitors can function to normalize the altered BM vascular permeability [82]. NOS inhibitors have been developed for several applications, many of which are under clinical investigation. A specific example is the development of a peptide that mimics the endogenous inhibition function of caveolin-1 and selectively acts on endothelial NOS3 [132].

## Conclusion and future perspectives

Immunotherapy offers the possibility of more specific and less long-term toxic therapy in AML. The growing attention to new immunotherapy strategies together with a greater elucidation of the AML pathophysiology has led to understanding how the TME plays key roles in hindering therapeutic efficacy and in modulating toxicity.

Transcriptomic signatures have been recently used to stratify AML patients into immune-infiltrated and immune-depleted disease revealing critical differences in immune gene expression across age groups and molecular disease subtypes [5]. Of utmost importance, a novel precision medicine-based conceptual framework was described by evaluating the response to the CD123xCD3 DART flotetuzumab in relapsed/refractory AML patients. Specifically, T cell-targeting immunotherapy has been found to be beneficial in subgroups of patients with immune-infiltrated TME. Furthermore, since flotetuzumab increases expression of PD-L1 in AML blast, there is strong rationale for conceiving clinical studies with sequential flotetuzumab and ICIs in AML [94]. Immunological stratification of pre-treatment BM samples combined with cytogenetic and mutational information may define AML patients who will potentially have greatest benefit from immunotherapies.

The development of preclinical models that faithfully recapitulate the TME of the human AML remains instrumental to dissect the mechanism underlining the formation of the TME and to test novel therapeutic approaches. Artificial 3D microenvironment recreating the topology of BM with the stromal and vascular niche components, such as BM-on-a-chip platforms, can offer a powerful tool [133]. Moreover, the in situ mapping of different subpopulations in the human BM would allow a better definition of the cell subsets involved and of stromal cell-specific markers that could lead to the development of selective stromal-targeted therapies [134, 135].

The future of immunotherapies foresees combinatorial strategies based not only on the possibility to increase targeting efficacy but also on the modulation of the immune escape mechanisms generated within the TME. Finally, precise identification of the immune escape mechanisms in individual AML patients will allow for personalized immunotherapy based on specific immune signatures.
